# Portal vein embolization, biembolization, and liver venous deprivation

**DOI:** 10.1590/0100-3984.2021.0040

**Published:** 2021

**Authors:** José Hugo Mendes Luz, Tiago Bilhim

**Affiliations:** 1 Department of Interventional Radiology, Curry Cabral Hospital, and NOVA Medical School, Universidade NOVA de Lisboa, Lisbon, Portugal.

*Dear editor*

We read with great interest the article “Liver venous deprivation prior to hepatectomy: an interventional radiology procedure”, authored by Alves et al.^(1)^, in a recent issue of **Radiologia Brasileira**. This is an excellent addition to the “Advances in Radiology” section of the journal, which highlights the latest developments in medical practice in Brazil. Liver regeneration prior to major hepatectomy is decisive in cancer management because it allows these potentially curative surgical procedures to be performed in otherwise inoperable patients, thus improving survival outcomes^(2)^. Portal vein embolization (PVE), used for decades as a method of inducing liver hypertrophy^(3)^, has recently been used in combination with embolization of one or more hepatic veins^(4)^.

**Figure 1:**
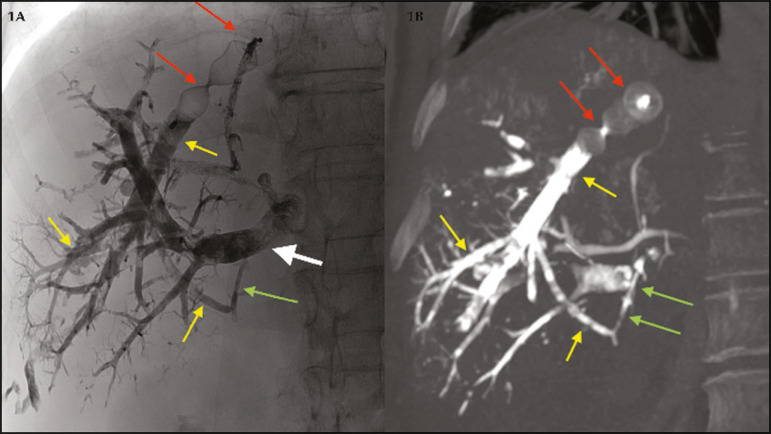
Fluoroscopic image (**1A**) obtained immediately after LVD and contrastenhanced coronal CT (**1B**) obtained 14 days after LVD. Note the vascular plug (red arrows) placed in the right hepatic vein for proximal embolization and NBCA plus lipiodol occluding the distal branches (yellow arrows). Note also NBCA plus lipiodol occluding a venovenous collateral (green arrows) and right portal vein embolization with NBCA plus lipiodol (white arrow).

Alves et al.^(1)^ described concomitant PVE and proximal right hepatic vein embolization with a vascular plug. This technique might be more appropriately designated biembolization^(5)^, being slightly different from liver venous deprivation (LVD). The LVD procedure has been described as: PVE plus proximal and distal embolization of the hepatic veins. Proximal embolization of the hepatic vein is accomplished with a vascular plug, as in biembolization, whereas distal embolization of the hepatic vein is achieved with N-butyl-cyanoacrylate (NBCA) plus lipiodol, as in LVD^(2)^. Why might this be relevant? Invariably, venovenous collaterals between liver segments V/VIII and IV are present^(6)^ and will increase in size after plug deployment^(4)^. Distal embolization with a liquid embolic agent (i.e., NBCA) not only eliminates flow in the target vein but also occludes those collaterals, which might have benefits in terms of liver hypertrophy induction. In addition, biembolization and LVD may require different technical approaches: LVD is usually performed through a percutaneous trans-hepatic approach^(4,7)^, making it easier to inject liquid embolic agents after plug deployment, whereas biembolization is performed through a transjugular approach (Figure 1).

Segment IV PVE, which was performed by Alves et al.^(1)^, has been reported to induce additional liver hypertrophy^(8)^. However, segment IV embolization is controversial: the segment IV portal branches are usually numerous and tiny, which increases the procedure time and the degree of technical difficulty; liquid embolic agents are trickier to use, because any reflux would cause nontarget embolization of liver segments II and III; due to the degree of technical difficulty, suboptimal embolization of segment IV might be an issue^(9)^; and segment IV is the main territory for systemic-portal venous shunts, possibly decreasing the efficacy of the procedure^(10)^. To overcome the limitations of PVE of segment IV, a more aggressive form of LVD has been proposed-extended LVD^(11)^-which consists of LVD plus middle hepatic vein embolization. Extended LVD has been shown to be safe and highly effective, promoting an unparalleled 53.4% increase in liver volume within only seven days^(11)^.

Future studies focusing on patient selection are needed. When and how to choose from such a variety of interventional tools? How to best predict post-hepatectomy liver failure? How can we choose between volumetric computed tomography and liver function studies (e.g., ^99m^Tc-mebrofenin hepatobiliary scintigraphy, gadoxetic acid-enhanced magnetic resonance imaging, and indocyanine green retention test)-or should we perform both? Most importantly, when is the liver ready for major surgery? How can we safely accelerate this preoperative process? Answering such questions are the reason for having multidisciplinary team meetings that allow personalized medical care, with input from different medical perspectives. We want to congratulate the authors not only for obtaining a regenerative outcome that allowed successful major hepatectomy within 41 days after embolization but also for highlighting the potential role and advantages of LVD versus PVE, providing grounds to expand future studies in this field^(12)^.
