# Supplementary material for: Portal vein embolization, biembolization, and liver venous deprivation
Source: Radiol Bras. 2021 May-Jun;54(3):206–7. doi: 10.1590/0100-3984.2021.0040 (PMC8177676; doi:10.1590/0100-3984.2021.0040)
Supplement: Related texts

# Reply

**Authors:** Vinicius de Pádua Vieira Alves, André Azevedo, Danilo Alves de Araujo, Leonardo Azevedo Marcondes Rodrigues, Feliciano Silva de Azevedo

**Affiliations:** 1 Universidade Federal Fluminense (UFF), Niterói, RJ, Brazil.; 2 Department of Interventional Radiology, Americas Medical City, Rio de Janeiro, RJ, Brazil.

We received with great enthusiasm the Letter to the Editor “Portal vein embolization, biembolization, and liver venous deprivation”, authored by Dr. Luz and Dr. Bilhim. We certainly agree that the precise nomenclature for the procedure described in our paper should be “Portal vein embolization with hepatic vein biembolization”. The added technique of distal embolization of the hepatic veins, described in liver venous deprivation, would likely represent further liver hypertrophy and probably better hepatic functionality than those achieved in the case we described. Our group has now standardized liver venous deprivation with a transhepatic approach as the technique of choice in such cases. In regard to the issue of hepatic segment IV embolization, we are of the same opinion (that it presents a technical challenge to the PVE procedure), and extended LVD with middle hepatic vein, rather than segment IV embolization, is now the preferred method in our department. Nevertheless, the theme of combined PVE and LVD (or biembolization) raises multiple questions and concerns, which will likely be addressed by prospective multicenter studies and collaborative multidisciplinary discussions to optimize medical care for the affected patients. We want to thank the authors for their interest in our paper, as well as for the perfectly highlighted issues, which further elevate the level of scientific debate in the area of interventional radiology.
